# Supplementary material for: Surface photogalvanic effect in Ag2Te
Source: Nat Commun. 2024 Jul 5;15:5651. doi: 10.1038/s41467-024-49576-4 (PMC11226672; doi:10.1038/s41467-024-49576-4)
Supplement: Supplementary file 1 — Supplementary Information [file 41467_2024_49576_MOESM1_ESM.pdf]

Supplementary Information for

Surface photogalvanic effect in Ag_2Te

Xiaoyi Xie[1,2#], Pengliang Leng[1,2#], Zhenyu Ding[3#], Jinshan Yang[4#], Jingyi Yan[4], Junchen Zhou[1,2], Zihan Li[1,2], Linfeng Ai[1,2], Xiangyu Cao[1,2], Zehao Jia[1,2],Yuda Zhang[1,2], Minhao Zhao[1,2], Wenguang Zhu[3,5,6], Yang Gao[5*], Shaoming Dong[4], Faxian Xiu[1,2,7,8,9*]

[1] State Key Laboratory of Surface Physics and Department of Physics, Fudan University, Shanghai 200433, China

[2] Shanghai Qi Zhi Institute, 41st Floor, AI Tower, No. 701 Yunjin Road, Xuhui District, Shanghai 200232, China

[3] International Center for Quantum Design of Functional Materials (ICQD), Hefei National Research Center for Physical Sciences at the Microscale, University of Science and Technology of China, Hefei 230026, China

[4] State Key Laboratory of High Performance Ceramics and Superfine Microstructure, Shanghai Institute of Ceramics, Chinese Academy of Science, Shanghai 200050, China

[5] Department of Physics, University of Science and Technology of China, Hefei 230026, China

[6] Hefei National Laboratory, Hefei 230088, China

[7] Institute for Nanoelectronic Devices and Quantum Computing, Fudan University, Shanghai 200433, China

[8] Zhangjiang Fudan International Innovation Center, Fudan University, Shanghai 201210, China

[9] Shanghai Research Center for Quantum Sciences, Shanghai 201315, China

[#] These authors contributed equally to this work

[*] Correspondence and requests for materials should be addressed to F. X. (E-mail: Faxian@fudan.edu.cn) and Y. G. (E-mail: ygao87@ustc.edu.cn)

This PDF file includes:

Supplementary Figures 1-10

Supplementary Notes 1-8

1. *I-V* characteristics and bias-dependent photoresponse in Ag_2Te
2. The two possible orientations of **b**-axis
3. Reproducibility of the turn-over experiment
4. Linear polarization anisotropy and scanning photocurrent at different wavelengths
5. The vanished response of β_{xxy}
6. Elimination of strain effect
7. Temperature dependence of the photocurrent
8. Theoretical calculation of shift current

Supplementary References 1-16

Supplementary Figures

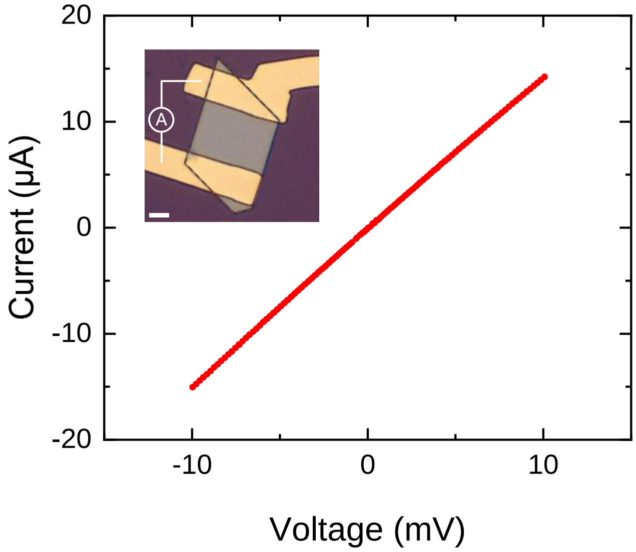

Supplementary Figure 1 | *I-V* characteristic of the Ag_2Te device shown in Fig. 1c of the main text. The scale bar is 5μm.

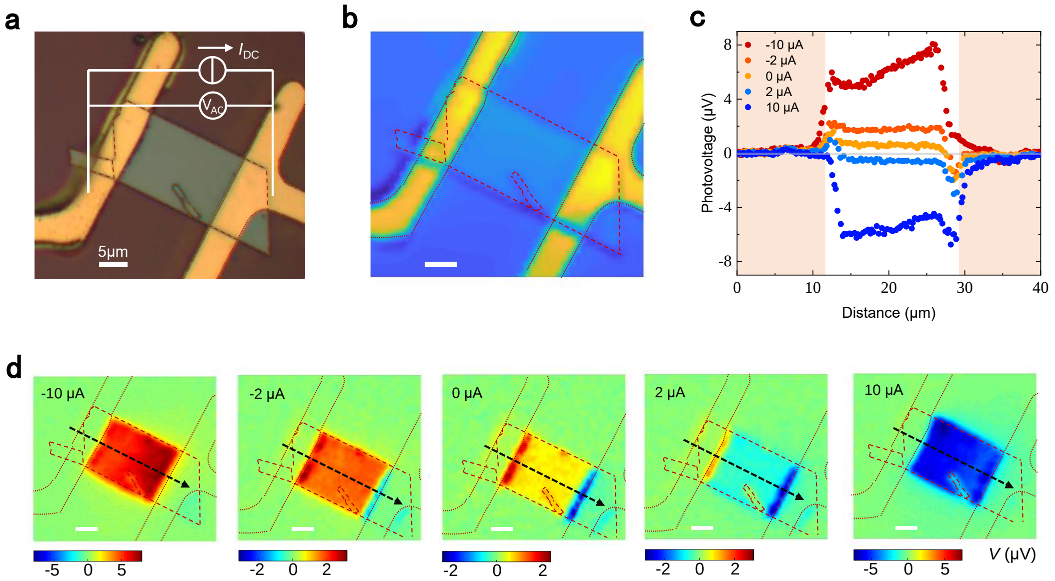

Supplementary Figure 2 | Bias current dependence of the photovoltage response in Ag_2Te. a,b, Optical image (**a**) and reflection image (**b**) of the Ag_2Te device. **c**, Line-profile of the photovoltage with different bias currents extracted from **d** along the black arrows. The two orange-pink regions indicate the electrodes. **d**, Photovoltage mapping with different bias currents. The laser wavelength is 690nm and the power is ~2.4uW. The scale bars are all 5μm.

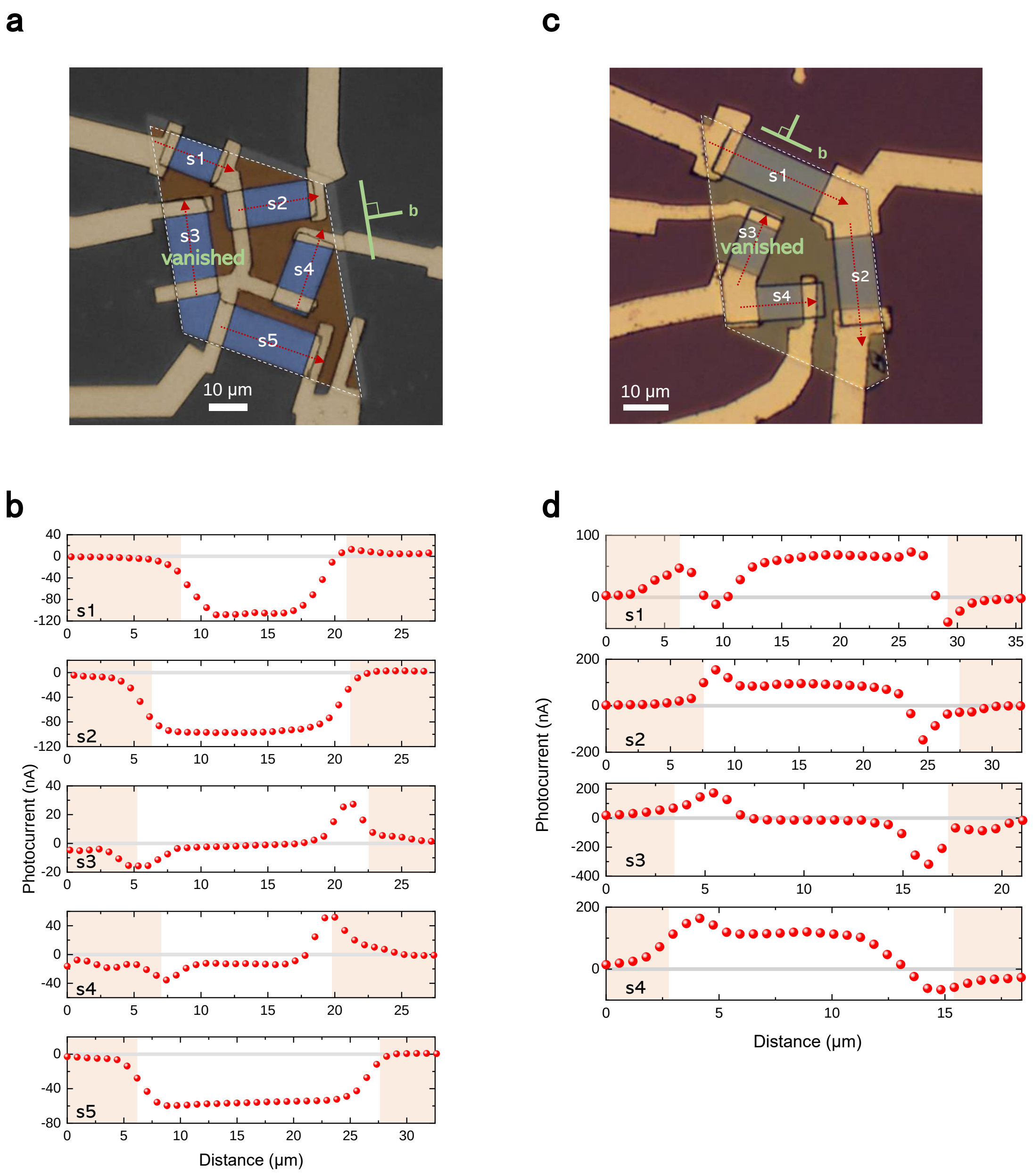

Supplementary Figure 3 | Photocurrent in Ag_2Te nanoplates with different b-axis orientations. a,b, Photocurrent in a nanoplate where **b**-axis is perpendicular to the edge, copied from Fig.2 of the main text. The photocurrent in s3 is vanished, which is parallel to the edge. **c,d,** Photocurrent in a nanoplate where the **b**-axis is parallel to the edge. The photocurrent in s3 is vanished, which is perpendicular to the edge. The red dotted arrows in **a**,**c** mark the directions of the measurements in **b**,**d**. The original shapes of the nanoplates are outlined by white dotted lines in **a**,**c**. The green axes in **a,c** denote the **b**-axis. The orange-pink shaded areas in **b,d** represent the electrode regions.The scale bars are 10μm. The laser wavelength is 690nm (**c,d**) and the power is ~100μW (**c**) and ~70μW (**d**).

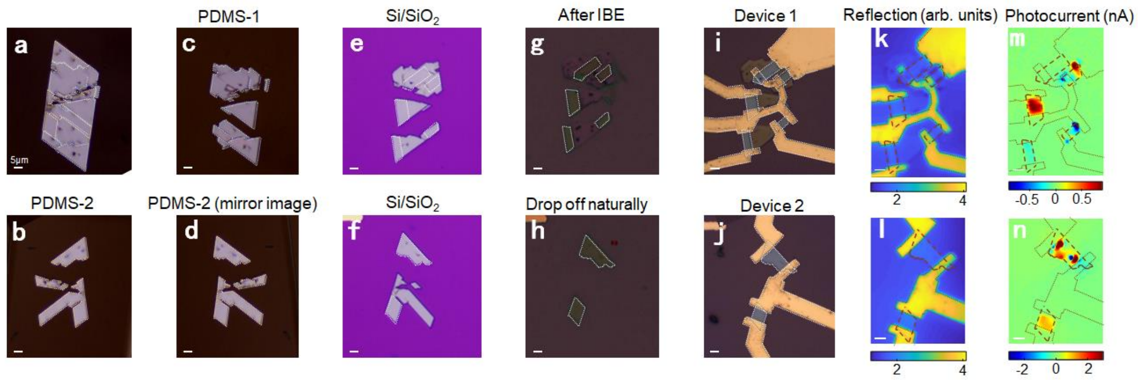

Supplementary Figure 4 | Detailed fabrication procedures of the turn-over device shown in Figs. 3e-k. a, The initial Ag_2Te nanoplate on PDMS-1, which was cut off by a probe. Then PDMS-2 was pressed onto the nanoplate, causing it to fragment, and then lift up with parts of the fragments. **b,c,** The fragments transferred to PDMS-2 (**b**) and remained on PDMS-1 (**c**). **d,** The mirror image of **b** shown for convenience. **e,f,** The two parts of fragments transferred onto Si/SiO_2 substrates. **g,** The fragments in **e** which were etched into 5 samples using ion beam etching (IBE). **h,** The fragments from **f**, with some naturally dropped off. **i,j,** The two devices fabricated from **g** and **h,** respectively. **k-n**, The reflection images (**k,l**) and photocurrent mappings (**m,n**) of these devices. The laser wavelength is 690nm and the power is ~80uW in **k-n**. The scale bars are 5µm.

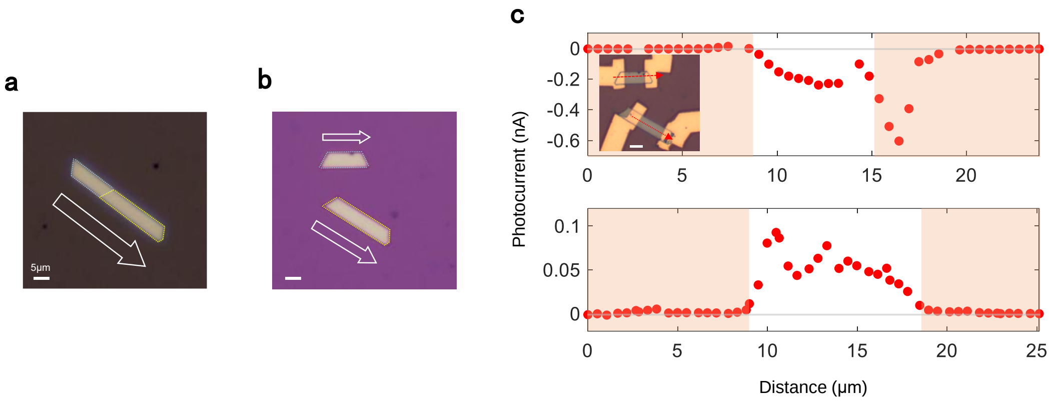

Supplementary Figure 5 | Turn-over test of a ribbon shape Ag_2Te nanoplate. a, Optical image of the initial Ag_2Te nanoplate on PDMS. **b**, Two parts of the nanoplate with different sides up. The white arrows in **a** and **b** indicate the same crystal orientation. **c,** Main: photocurrent distribution in the two samples along the red arrows in the inset. Orange regions indicate the electrodes. Inset: optical image of the devices. The laser wavelength is 690nm and the power is ~2.4uW. The scale bars are 5µm.

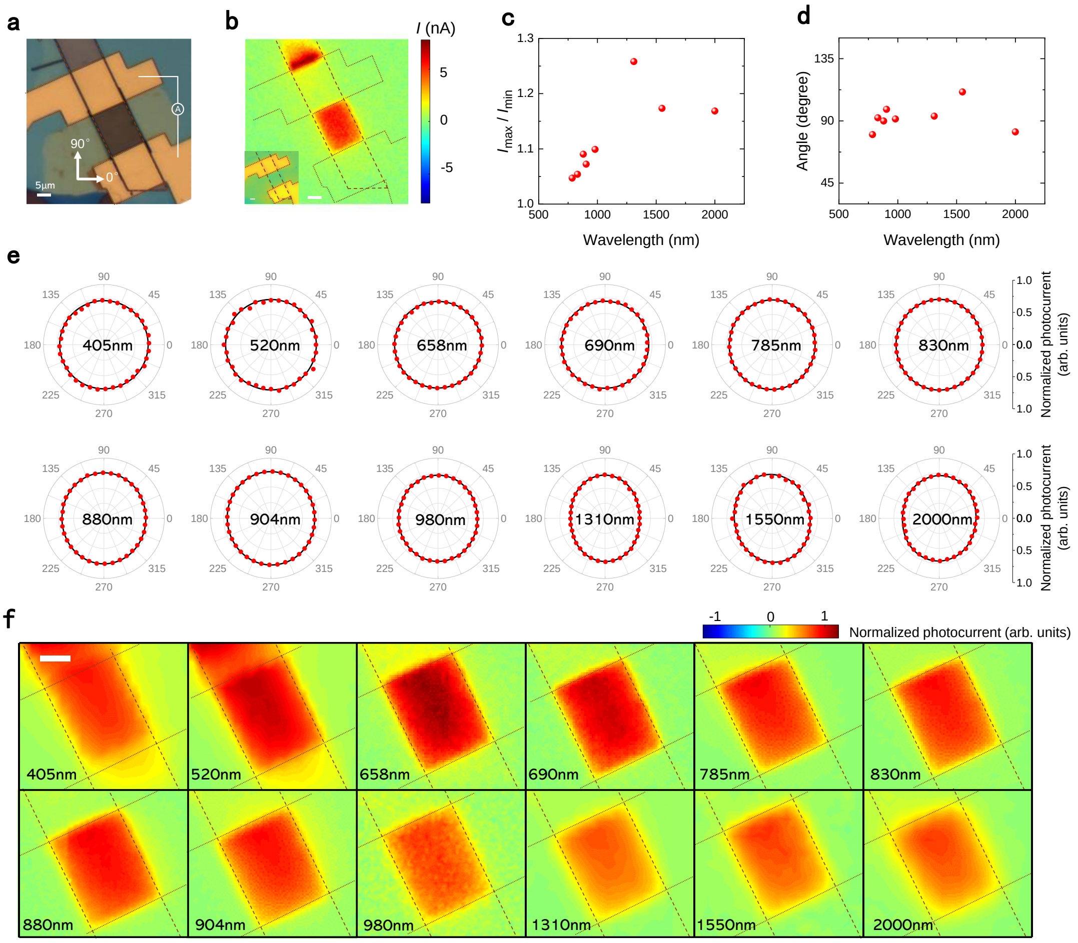

Supplementary Figure 6 | Linear polarization dependence and photocurrent mapping with varied wavelength from 405nm to 2000nm in Ag_2Te. a, Optical image of an hBN-Ag_2Te-hBN device. The light green region is the top hBN layer, covering the whole channel. **b**, Photocurrent mapping (main) and corresponding reflection image (inset) of the device under a 690-nm illumination. **c**,**d**, The anisotropy ratio (**c**) defined as I_{max}/I_{min} and the orientation of polarization (**d**) of 690~2000nm. **e**,**f**, Linear polarization dependence (**e**) and photocurrent mapping (**f**) of 405~2000nm. The black curves of 405~690nm in (**e**) are constants and the cases of 785~2000nm are the fitted results with sinusoidal functions. The scale bars are all 5μm.

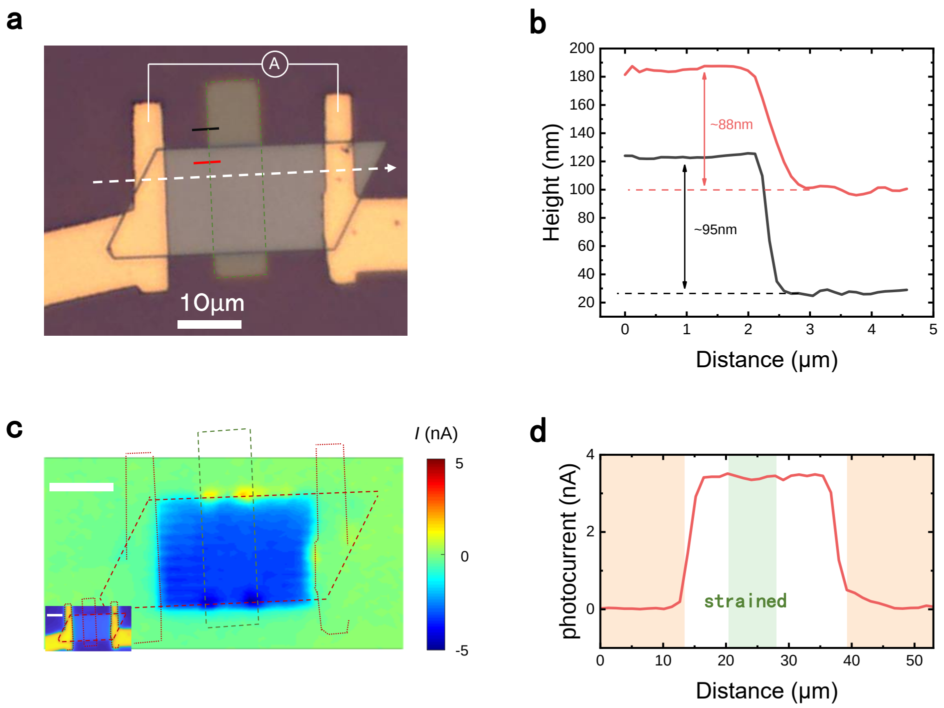

Supplementary Figure 7 | The effect of artificially applied strain. a,c, Optical image (**a**), reflection image (inset of **c**), and photocurrent mapping (**c**) of the strain device. **b,** Depth of the groove measured by atomic force microscopy (AFM). The two lines are marked in **a** colored as black and red, respectively. **d**, The photocurrent distribution along the white arrow indicated in **a**. The laser wavelength is 690nm and the power is ~7μW. The scale bars are 10μm.

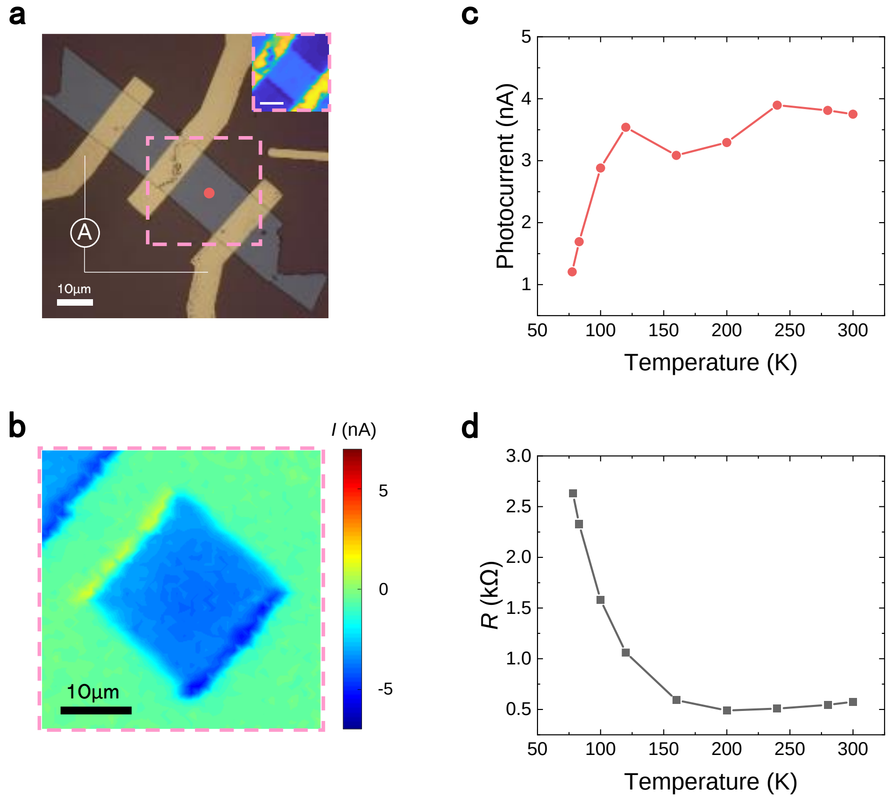

Supplementary Figure 8 | Temperature dependence of the photocurrent. a-b, Optical image (**a**), photocurrent mapping at room temperature (**b**), and corresponding reflection image (inset of **a**). **c**, Temperature dependence of the photocurrent (absolute value). The data is obtained with illumination at the center of the device marked by the red spot in **a**. **d**, Temperature dependence of the two-terminal resistance. The laser wavelength is 690nm and the power is ~14 μW. The scale bars are all 10μm.

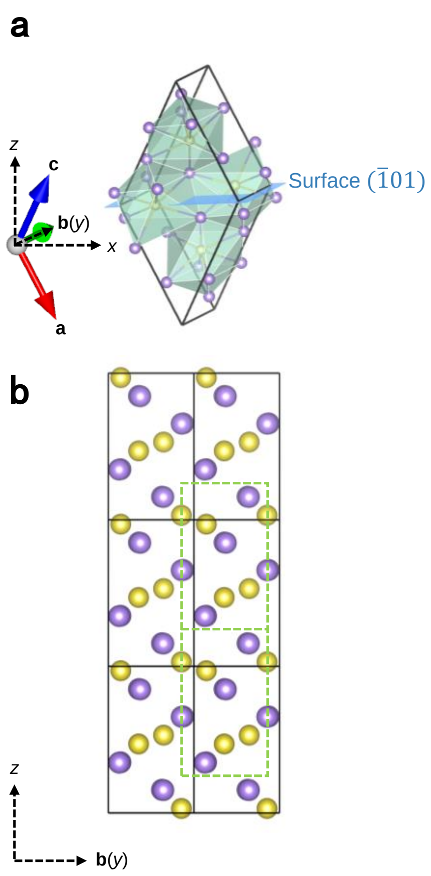

Supplementary Figure 9 | Schematic of the slab model. a, Unit cell of Ag_2Te. The blue plane $(\bar{1}01)$ indicates the surface orientation of Ag_2Te nanoplate. **b**, Side view of the bulk structure. **c**, The slab model with a vacuum layer. The green dotted boxes in **b**,**c** show the extracted atom makeup of the slab.

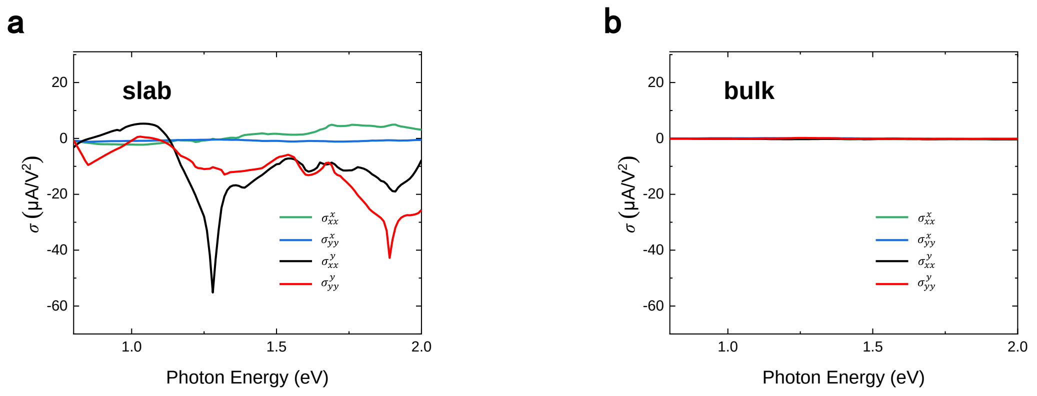

Supplementary Figure 10 | Calculated shift current in the slab (a) and bulk (b) of Ag_2Te.

Supplementary Notes

Supplementary Note 1: *I-V* characteristics and bias-dependent photoresponse in Ag_2Te

A linear dependence of dark current on bias voltage (see Supplementary Figure 1) in Ag_2Te device demonstrates good Ohmic contacts between Ag_2Te and electrodes, indicating the absence of distinct junction structures accounting for the photocurrent response. Besides, the two-terminal resistance of the device is just ~660Ω, which is attributed to the low resistivity of Ag_2Te resulting from its narrow bandgap. This characteristic sets it apart from traditional BPVE materials with wider bandgaps. As a result, it's challenging to investigate the dc *I-V* characteristic in Ag_2Te with laser on owing to a large dark current.

Nevertheless, we measured the net photovoltage response with a dc bias current using a lock-in amplifier. Another Ag_2Te device with a similar two-terminal structure is fabricated for this measurement and the result is shown in Supplementary Figure 2. The photovoltage with 0μA bias current exhibits a relatively weaker positive SPGE response distributed over the device and a stronger Schottky junction-induced PV near the contacts. The response across the device can be strengthened or weakened, and even changes sign by applying an opposite bias current, because the applied electric field has a similar effect on the device as SPGE. As for the regions near the contacts, the measured response is a combination of the three mechanisms: SPGE, PV induced by the Schottky junction, and PV induced by the applied electric field.

Supplementary Note 2: The two possible orientations of b-axis

As we stated in the main text, there are two possible in-plane orientations of **b**-axis: perpendicular or parallel to one of the edges. It can be clearly demonstrated in the crystallographic orientation dependence of SPGE.

According to the symmetry analysis, the photocurrent perpendicular to **b**-axis would vanish under unpolarized light. Therefore, the **b**-axis in the nanoplate shown in Fig. 2 of the main text (or Supplementary Fig. 3a-b here) is perpendicular to one of the edges. The same experiment performed in some other nanoplates displays another case, as shown in Supplementary Fig. 3c-d. The photocurrent vanishes in s3, which indicates the **b**-axis aligns with one of the edges.

Supplementary Note 3: Repeatability of the turn-over experiment

A ribbon-shaped Ag_2Te nanoplate, as depicted in Supplementary Fig. 5a, was cut into two samples, and one of them was flipped over using a tip. Subsequently, the two samples were transferred onto a Si/SiO_2 substrate, as shown in Supplementary Fig. 5b, and fabricated into devices, as indicated in the inset of Supplementary Fig. 5c. Photocurrent distribution of the two samples was measured as shownin Supplementary Fig. 5c. While the upper sample exhibits a strong PV and/or PTE response at the right

contact, the region of Ag_2Te still demonstrates a typical SPGE response. The photocurrent directions of the two samples are observed to be opposite in comparison to the crystallographic orientation indicated by the white arrows in Supplementary Fig. 5a-b, which supports our hypothesis regarding the surface origin.

Supplementary Note 4: Linear polarization anisotropy and scanning photocurrent at different wavelengths

Supplementary Figure 6 shows the thickest sample exhibited in Fig. 4a with a thickness of ~646nm. This sample is fabricated into a sandwich structure with a top hBN of ~19nm and a bottom hBN of ~25nm. These repeatable results on the hBN-Ag_2Te-hBN structure clarify SPGE as an intrinsic response in Ag_2Te irrespective of the environment.

The spectral performance presented in Fig. 4b is also obtained from this particular device. We demonstrate the polarization dependence and photocurrent mapping of the device when illuminated with wavelengths ranging from 405 nm to 2000 nm. As depicted in Supplementary Fig. 6f, a typical BPVE response is observed across the entire spectral range. Additionally, linear polarized anisotropy emerges beyond 690 nm, indicating variations in the relative magnitudes of β_{yxx} and β_{yyy}.

However, performing photocurrent mapping under mid-infrared conditions (3870 nm, 4560 nm, and 10600 nm) poses challenges. Therefore, the results shown in Fig. 4b for the mid-infrared range were obtained by averaging five points near the center of the sample.

Supplementary Note 5: The vanished response of $\boldsymbol{\beta_{xxy}}$

We did not observe any response from β_{xxy} in Ag_2Te, which is consistent with the findings in Zhang's work on monolayer WSe_2 (not notable)[1] and Dong's work on 3R-MoS_2 (notable but still small)[2]. The response is considered too weak to be measured, but it could potentially be enhanced by further reducing the symmetry.

It should be noted that the symmetry constraints obtained from the phenomenological description Eq. (1) shown in the main text are general but the detailed microscopic processes could further restrict the photocurrent generation[3]. Among the symmetry constraints-allowed structure, it's still possible to derive a negligible β with a relatively high symmetry or a negligible component of β along a relatively high symmetric orientation. Another possibility accounting for the tiny response is that the present shift current theory ignores the recombination process[2], which could give rise to an opposite shift that cancels out with the shift during excitation[4].

On the other hand, it is conceivable that the response could be unusually large within the further reduced symmetry due to specific factors. The presence of electric polarization, sensitivity to atomic displacement[2], newly proposed mechanisms like wall-to-wall charge shift[5], and other undiscovered mechanisms may contribute to this enhancement.

Supplementary Note 6: Elimination of strain effect

In principle, strain could break both screw rotation symmetry C_{2b} and the glide mirror symmetry $\widetilde{M_b}$ in Ag_2Te, while the inversion symmetry is stable towards strain. Alternatively, several randomly distributed strains can certainly break the inversion symmetry. However, the distortion within the Ag_2Te nanoplate is intricate in this case. Therefore, the photocurrent distribution (both sign and magnitude) would be also intricate and differ from our results which are highly uniform shown in the photocurrent mapping.

In addition, another possible approach to break the inversion symmetry is the strain gradient, referred to flexo-photovoltaic effect, which could be introduced by stacking or AFM tip[6,7]. However, the mapping results also confirm that the strain gradient should be uniform. A uniformly distributed strain gradient covering the Ag_2Te nanoplate is nearly impossible to emerge spontaneously in a series of samples.

Meanwhile, we performed an experiment to verify the effect of strain. We artificially applied strain on the Ag_2Te nanoplate employing a groove structure (referring to the 3R-MoS_2 work[8]) as shown in Supplementary Figure 7. The SiO_2/Si substrate was pre-patterned by reactive ion etching (RIE) to form a groove with a depth of ~95 nm, outlined by the green dashed rectangle in Supplementary Figure 7a,c. The Ag_2Te nanoplate was dry-transferred onto the substrate and pushed down to the bottom of the groove by PDMS. The altitude of Ag_2Te stepped over the groove was measured to confirm that the nanoplate was completely pushed down (Supplementary Figure 7b). The uniformly distributed strain would be applied in the center region of the groove.

The photocurrent mapping is shown in Supplementary Figure 7c-d, which is rather uniform over the whole nanoplate. There is no evident difference within and out of the groove region, suggesting there is no evident enhancement to the photocurrent in Ag_2Te of uniaxial strain. The result is expected, as we discussed above that strain couldn't break the inversion symmetry.

Supposing that the photocurrent in Ag_2Te is induced by some kind of unknown strain, the appropriate orientation of the strain should be consistent with the photocurrent. Accordingly, the geometry (relative orientation between the groove and Ag_2Te) of the device here is sufficient for checkout. Now that the artificially applied strain cannot adjust the photocurrent in Ag_2Te, the photocurrent itself is unlikely induced by strain either.

Supplementary Note 7: Temperature dependence of the photocurrent

As shown in Supplementary Figure 8c, the photocurrent slightly fluctuates with the temperature decreasing from 300K to 120K, whereas a sharp drop emerges from 120K to 78K. In Ag_2Te, the absorption under visible illumination is unlikely to be critically

affected by tuned bandgap at different temperatures, especially the excitation taking place in the surface state. On the other hand, the two-terminal resistance of the device was also measured, which demonstrates a typical semiconductor behavior (Supplementary Figure 8d), opposite to the photocurrent. The drop in measured photocurrent is attributed to the effect of increased resistance, which influences the diffusion of the photocurrent. Similar temperature dependence is common in previous BPVE works[1,9]. We should emphasize that the photocurrent is generated at the surface of Ag_2Te. However, the contribution of resistance is global (from surface, bulk, and contacts) because the whole device participates in propagating the photocurrent.

Supplementary Note 8: Theoretical calculation of shift current

8.1 Structural modeling:

To analyze the bulk photovoltaic effect (BPVE, or rather surface photogalvanic effect (SPGE)) of Ag_2Te along the $(\bar{1}01)$ plane, we constructed a slab model from the Ag_2Te crystal as shown in Supplementary Figure 9c, with both the top and bottom surfaces oriented along the $(\bar{1}01)$ plane. The process of slab extraction from the expanded unit cell is detailed in Supplementary Fig. 9, showing the alignment of the slab with the original crystal axes. A vacuum layer has been added along the **c**-axis of the slab model to eliminate interactions from periodic boundaries in this direction. We would use the slab model to represent an isolated surface structure in the following calculation.

8.2 Electronic structure calculations:

We performed electronic structure calculations using the Vienna Ab initio Simulation Package (VASP), adopting the Perdew-Burke-Ernzerhof (PBE) form of the generalized gradient approximation (GGA) for the exchange-correlation functional[10–12]. The kinetic energy cutoff for the plane-wave basis was set to 400 eV. For structural optimization and static self-consistent calculations, the Brillouin zone was sampled using Monkhorst-Pack **k**-point meshes of 9×17×1 and 15×27×1, respectively. The positions of the atoms were fully optimized with a force convergence criterion of -0.01eV/Å. To facilitate high-precision optical calculations, the energy convergence criterion for the electronic self-consistency was set to 10-7 eV. Additionally, a dipole correction was applied in the out-of-plane direction.

8.3 Calculation of nonlinear optical conductivity:

We utilize the Shift Current (SC) framework to address issues related to the BPVE (SPGE), which mainly describes the interband absorption. Considering the electric field component of a monochromatic light, it can be expressed as:

$$\boldsymbol{E}(t) = \boldsymbol{E}(\omega)e^{-i\omega t} + \boldsymbol{E}(-\omega)e^{i\omega t}, \quad (1)$$

where E represents the alternating current (AC) electric field of the light.

As a second-order optical response, the SC describes the direct current (DC) photocurrent output in the material's a-direction due to light polarized along the b-direction, expressed as:

$$J^a = \sigma^a_{bb}(\omega)\mathrm{Re}\,[E_b(\omega)E_b(-\omega)], \quad (2)$$

where $\sigma^a_{bb}(\omega)$ represents the nonlinear optical conductivity induced by the SC, derived from Kubo formula. Note that σ^a_{bb} and the measured coefficient β_{abb} in the main text can be converted with each other according to $\beta_{abb} = 2\sigma^a_{bb}/(\varepsilon_0 c)$, where ε_0 is the vacuum permittivity and c is the speed of light. $\sigma^a_{bb}(\omega)$ can be expressed as[13]:

$$\sigma^a_{bb}(\omega) = -\frac{i\pi g_s e^3}{\hbar^2}\int [d\mathbf{k}] \sum_{nm} f_{nm} r^b_{mn} r^{b;a}_{nm} \times [\delta(\omega_{mn} - \omega) + \delta(\omega_{nm} - \omega)], \quad (3)$$

Here, g_s denotes the spin degeneracy, $f_{nm} = f_n - f_m$ and $\hbar\omega_{nm} = \varepsilon_n - \varepsilon_m$ are the differences in occupation numbers and energy eigenvalues between bands indexed by n and m at point **k**, respectively (**k** is omitted for simplicity). r^b_{mn} and $r^{b;a}_{nm}$ represent the interband dipole and its 'generalized derivative', which can be constructed from the Berry Connection $A^a_{\mathbf{k}nm} = i\langle u_{\mathbf{k}n} \mid \partial_a u_{\mathbf{k}m}\rangle$, as:

$$r^a_{\mathbf{k}nm} = (1 - \delta_{nm}) A_{\mathbf{k}nm},$$

$$r^{a;b}_{\mathbf{k}nm} = \partial_b r^a_{\mathbf{k}nm} - i\left(A^b_{\mathbf{k}nn} - A^b_{\mathbf{k}mm}\right) r^a_{\mathbf{k}nm}, \quad (4)$$

where $|\partial_a u_{\mathbf{k}m}\rangle$ denotes the cell-periodic part of the Bloch eigenstate, and ∂_a is shorthand for ∂/∂_{k_a}.

It is crucial to note that, in calculations involving slab models, excluding the impact of the vacuum layer is essential for accurately determining the material's effective shift current output, formalized as:

$$\sigma^{a(\text{eff})}_{bb} = \frac{l}{l_a}\sigma^a_{bb}, \quad (5)$$

Where l represents the total thickness of the slab along the out-of-plane direction, and l_a denotes the material's effective layer thickness. Specifically, for our slab under discussion, $l = 38.59$Å and $l_a = 13.26$Å. For simplicity, the superscript 'eff' is omitted in subsequent diagrams and descriptions.

We implemented interpolation in k-space using the method of maximally localized Wannier functions (MLWFs), facilitated by the WANNIER90 code package[14,15]. This technique was specifically employed to improve the convergence of SC conductivity calculations in **k**-space. The interpolated **k**-point grid was expanded to 500×500×1. To specifically address the electronic properties near the Fermi level, trial orbitals for the Wannier projection were chosen as Ag-s and Te-p. The calculations of the aforementioned SC conductivity were conducted using the postw90-berry-sc module in the wannier90 post-processing program (postw90.x)[16].

The photon energy-dependent $\sigma^a_{bb}(\omega)$ in the slab across different in-plane components are shown in Supplementary Figure 10a (with Cartesian directions x and y labeled in the figure). The non-zero terms are σ^y_{xx} and σ^y_{yy}, both maintaining consistent signs and trends within the energy windows. The tiny value in σ^x_{xx} is

attributed to algorithmic errors. For comparison, similar calculations were performed for bulk Ag_2Te (Supplementary Figure 10b), where all components consistently remain zero.

The calculation shows identical results with the qualitative symmetry analysis that a second-order nonlinear response along the **b**-axis exists on the surface of Ag_2Te, while prohibited in the bulk. The resembled σ_{xx}^{y} and σ_{yy}^{y} also predict a unipolar and relatively weak polarization dependence of the measured photocurrent. Phenomenologically, the calculation captures the symmetry and polarization characteristics in the observed SPGE (Fig. 2 of the main text).

On the other hand, we didn't observe the calculated peak near 1.28eV and 1.89eV. There is probably no such practical laser wavelength (Fig. 4b of the main text) that completely coincides with the predicted energy. Moreover, the prominent contribution from specific transition is likely covered in the more complex actual band structure, especially within the large energy range.

Supplementary References

1. Zhang, Y. J. *et al.* Enhanced intrinsic photovoltaic effect in tungsten disulfide nanotubes. *Nature* **570**, 349–353 (2019).
2. Dong, Y. *et al.* Giant bulk piezophotovoltaic effect in 3R-MoS_2. *Nature Nanotechnol.* **18**, 36–41 (2023).
3. Ibañez-Azpiroz, J., Souza, I. & de Juan, F. Directional shift current in mirror-symmetric BC_2 N. *Phys. Rev. Research* **2**, 013263 (2020).
4. Sturman, B. I. Ballistic and shift currents in the bulk photovoltaic effect theory. *Phys.-Usp.* **63**, 407–411 (2020).
5. Kim, B., Park, N. & Kim, J. Giant bulk photovoltaic effect driven by the wall-to-wall charge shift in WS_2 nanotubes. *Nature Commun.* **13**, 3237 (2022).
6. Jiang, J. *et al.* Flexo-photovoltaic effect in MoS_2. *Nat. Nanotechnol.* **16**, 894–901 (2021).
7. Yang, M.-M., Kim, D. J. & Alexe, M. Flexo-photovoltaic effect. *Science* **360**, 904–907 (2018).
8. Dong, Y. *et al.* Giant bulk piezophotovoltaic effect in 3R-MoS_2. *Nature Nanotechnol.* **18**, 36–41 (2023).
9. Akamatsu, T. *et al.* A van der Waals interface that creates in-plane polarization and a spontaneous photovoltaic effect. *Science* **372**, 68–72 (2021).
10. Kresse, G. & Furthmüller, J. Efficient iterative schemes for *ab initio* total-energy calculations using a plane-wave basis set. *Phys. Rev. B* **54**, 11169–11186 (1996).
11. Perdew, J. P., Burke, K. & Ernzerhof, M. Generalized Gradient Approximation Made Simple. *Phys. Rev. Lett.* **77**, 3865–3868 (1996).
12. Blöchl, P. E. Projector augmented-wave method. *Phys. Rev. B* **50**, 17953–17979 (1994).
13. Sipe, J. E. & Shkrebtii, A. I. Second-order optical response in semiconductors. *Phys. Rev. B* **61**, 5337–5352 (2000).
14. Marzari, N., Mostofi, A. A., Yates, J. R., Souza, I. & Vanderbilt, D. Maximally localized Wannier functions: Theory and applications. *Rev. Mod. Phys.* **84**, 1419–1475 (2012).
15. Pizzi, G. *et al.* Wannier90 as a community code: new features and applications. *J. Phys.: Condens. Matter* **32**, 165902 (2020).
16. Ibañez-Azpiroz, J., Tsirkin, S. S. & Souza, I. *Ab initio* calculation of the shift photocurrent by Wannier interpolation. *Phys. Rev. B* **97**, 245143 (2018).
